# Real-world retrospective cohort study ARCTIC shows burden of comorbidities in Swedish COPD versus non-COPD patients

**DOI:** 10.1038/s41533-018-0101-y

**Published:** 2018-09-10

**Authors:** Björn Ställberg, Christer Janson, Kjell Larsson, Gunnar Johansson, Konstantinos Kostikas, Jean-Bernard Gruenberger, Florian S. Gutzwiller, Leif Jorgensen, Milica Uhde, Karin Lisspers

**Affiliations:** 1 Department of Public Health and Caring Sciences, Family Medicine and Preventive Medicine, Uppsala University, Uppsala, Sweden; 2 Department of Medical Sciences: Respiratory, Allergy and Sleep Research, Uppsala University, Uppsala, Sweden; 3 Department of Pulmonary Medicine, Work Environment Toxicology, Karolinska Institutte, SE-171 77, Stockholm, Sweden; 4 Novartis Pharma AG, Basel, Switzerland; 5 IQVIA, Copenhagen, Denmark; 6 IQVIA, Solna, Sweden

## Abstract

This study aimed to generate real-world evidence to assess the burden of comorbidities in COPD patients, to effectively manage these patients and optimize the associated healthcare resource allocation. ARCTIC is a large, real-world, retrospective cohort study conducted in Swedish COPD patients using electronic medical record data collected between 2000 and 2014. These patients were studied for prevalence of various comorbidities and for association of these comorbidities with exacerbations, mortality, and healthcare costs compared with an age-, sex-, and comorbidities-matched non-COPD reference population. A total of 17,479 patients with COPD were compared with 84,514 non-COPD reference population. A significantly higher prevalence of various comorbidities was observed in COPD patients 2 years post-diagnosis vs. reference population, with the highest percentage increase observed for cardiovascular diseases (81.8% vs. 30.7%). Among the selected comorbidities, lung cancer was relatively more prevalent in COPD patients vs. reference population (relative risk, RR = 5.97, *p* < 0.0001). Ischemic heart disease, hypertension, depression, anxiety, sleep disorders, osteoporosis, osteoarthritis, and asthma caused increased mortality rates in COPD patients. Comorbidities that were observed to be significantly associated with increased number of severe exacerbations in COPD patients included heart failure, ischemic heart disease, depression/anxiety, sleep disorders, osteoporosis, lung cancer, and stroke. The cumulative healthcare costs associated with comorbidities over 2 years after the index date were observed to be significantly higher in COPD patients (€27,692) vs. reference population (€5141) (*p* < 0.0001). The data support the need for patient-centered treatment strategies and targeted healthcare resource allocation to reduce the humanistic and economic burden associated with COPD comorbidities.

## Chronic lung disease: Tailoring treatment to encompass co-existing conditions

Co-existing conditions should be taken into consideration when treating patients with chronic lung disease to ensure coherent and cost-effective disease management. In a large-scale study of the Swedish population, Björn Ställberg at Uppsala University and co-workers analyzed electronic medical records spanning fourteen years for 17,479 patients with chronic obstructive pulmonary disease (COPD) and compared their health status with 84,514 age-, sex- and comorbidity-matched non-COPD members of the population. Patients with COPD were significantly more likely to suffer from co-morbidities two years after initial diagnosis than their non-COPD counterparts, with cardiovascular diseases being the most common comorbidities. Lung cancer, hypertension, depression and sleep disorders were among other comorbidities more prevalent in the COPD population. These data support the need for fully integrated, targeted healthcare to reduce mortality and the economic burden associated with COPD.

## Introduction

Chronic obstructive pulmonary disease (COPD) is a common disease and it was estimated to affect 384 million persons in the year 2010, corresponding to a global prevalence of 11.7% (8.4%–15.0%).^1^ According to the 2010 Global Burden of Disease study, COPD has been estimated to be the third leading cause of mortality worldwide.^2^

COPD is also one of the leading causes of morbidity worldwide, and the morbidity is further affected by presence of concomitant chronic conditions.^3^ These chronic comorbidities play a potential role in the prognosis of COPD and health outcomes of patients. Evidence suggests that comorbidities increase the risk for exacerbations and hospitalizations, reduce health status, and increase the risk of mortality in COPD patients.^4–11^ Various comorbidities studied in patients with COPD include asthma; lung cancer; cardiovascular diseases, such as heart failure, ischemic heart disease, arrhythmias, peripheral vascular disease, and hypertension; osteoporosis; depression; anxiety; metabolic syndrome and diabetes; gastroesophageal reflux; bronchiectasis; and obstructive sleep apnea.^3,5,8,12–16^

Comorbidities are identified as major drivers of excess use of healthcare resource utilization (HCRU) and additional overall costs of managing COPD patients.^6,17^ A recently conducted study in the Danish general practice settings reported that comorbidities cause up to 28% variation in the healthcare expenditures, whereas other demographic characteristics, such as age and sex only cause 5% variation.^18^ These healthcare expenditures further increase with increase in the number of comorbid conditions.

Hence, it is important to generate real-world evidence to better understand the burden of comorbidities in COPD to effectively manage these patients and find ways to optimize the associated healthcare resource allocation. The aim of this study was therefore to provide real-world evidence on the burden of comorbidities in COPD in the Swedish primary care and identify areas for improvement and management of COPD patients.

## Results

Out of the identified 18,586 eligible patients with a COPD diagnosis listed in EMRs, 291 patients were excluded as they were diagnosed with COPD before 40 years of age. Following case-control matching, a total of 17,479 patients with COPD (ICD-10: J44.0) were studied under cases and these were compared with 84,514 age- and sex-matched control patients. Patient demographics of the COPD population and the age- and sex-matched reference population have been illustrated in the recent publication on economic burden assessed in the ARCTIC study.^19^

Patients in the COPD and reference populations with comorbidities, including hypertension, depression/anxiety, sleep disorders, lung cancer, osteoarthritis, and asthma were aged between 60 and 70 years, while populations with comorbidities, including heart failure, osteoporosis, and stroke had higher age distribution of 70–80 years (Table 1). Although a similar age distribution was seen between the comorbidity and reference groups for most comorbidities, significant differences were observed among patients with heart failure, hypertension, depression/anxiety, osteoarthritis, and asthma (Table 1). Further, significant differences by sex were observed between the groups with heart failure, hypertension, sleep disorders, osteoarthritis, and asthma as comorbidities (Table 1).

**Table 1:** Age and sex distribution of patients with comorbidities in COPD and reference groups (age at index date and comorbidities 2 years before)

Variable	*p*-value	COPD population with comorbidities	Reference population with comorbidities
*Heart failure*			
Age (years)	<0.0001	76.47 (9.90)	78.27 (9.99)
Sex (F/M)	0.0175	1079 (44.81)/1329 (55.19)	874 (48.50)/928 (51.50)
*Ischemic heart disease*			
Age (years)	0.1583	73.34 (10.06)	73.74 (10.49)
Sex (F/M)	0.5396	1063 (42.98)/1410 (57.02)	1261 (42.16)/1730 (57.84)
*Hypertension*			
Age (years)	0.0007	71.15 (10.07)	70.54 (10.68)
Sex (F/M)	0.0334	2934 (54.12)/2487 (45.88)	4725 (55.96)/3718 (44.04)
*Depression/anxiety*			
Age (years)	<0.0001	65.60 (12.01)	63.62 (12.15)
Sex (F/M)	0.5804	946 (66.57)/475 (33.43)	1406 (67.47)/678 (32.53)
*Sleep disorders*			
Age (years)	0.4421	64.52 (9.34)	64.02 (9.82)
Sex (F/M)	0.0245	153 (40.37)/226 (59.63)	252 (47.91)/274 (52.09)
*Osteoporosis*			
Age (years)	0.6458	75.24 (9.41)	74.96 (10.74)
Sex (F/M)	0.1146	449 (87.18)/66 (12.82)	550 (90.16)/60 (9.84)
*Lung cancer*			
Age (years)	0.2072	69.96 (8.25)	69.03 (9.82)
Sex (F/M)	0.6146	214 (50.00)/214 (50.00)	111 (52.11)/102 (47.89)
*Stroke*			
Age (years)	0.3910	74.81 (9.99)	75.29 (10.31)
Sex (F/M)	0.4874	222 (46.06)/260 (53.94)	476 (47.98)/516 (52.02)
*Osteoarthritis*			
Age (years)	<0.0001	70.82 (10.03)	68.20 (10.50)
Sex (F/M)	0.0087	595 (63.03)/349 (36.97)	1943 (67.68)/928 (32.32)
*Asthma*			
Age (years)	<0.0001	67.33 (12.24)	66.01 (11.96)
Sex (F/M)	0.0407	1681 (61.60)/1048 (38.40)	1817 (64.25)/1011 (35.75)

Mean (standard deviation) values presented for age, while *n* (%) values provided for gender

*F/M* females/males, *COPD* chronic obstructive pulmonary disease

### Prevalence of comorbidities

**Fig. 1:**
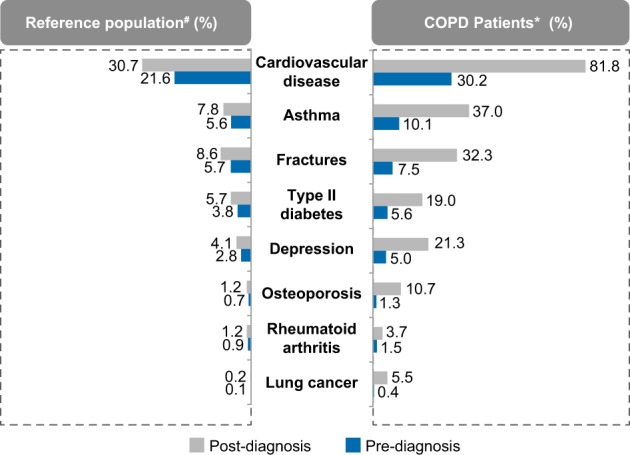
Prevalence of comorbidities (%) in COPD vs. reference population at 2 years pre- and post-first COPD diagnosis, #*N* = 52,208; *N = 13,052. COPD chronic obstructive pulmonary disease

A significantly higher prevalence of the selected comorbidities was observed in COPD patients compared with the reference population at 2 years pre- and post-first COPD diagnosis (*p* < 0.0001) (Fig. 1).

The prevalence of comorbidities increased in both groups after 2 years post-diagnosis. However, the percentage increase in the prevalence of comorbidities when calculated pre- and post-diagnosis was significantly higher in COPD patients compared with the reference population (*p* < 0.0001). The highest percentage increase was observed in the prevalence of cardiovascular diseases, which was 81.8% in COPD patients compared to 30.7% in the reference group (Fig. 1). The increase in the prevalence of comorbidities in the reference population might be attributed to the increase in age of the subjects.

Data showing >5-fold, >3-fold, and >2.5-fold increased prevalence of comorbidities in COPD population than the reference population are provided in the additional file (Tables S1, S2, and S3).

Among the nine selected comorbidities, lung cancer was more prevalent in patients with COPD than in the reference population (risk increased by 5.97), followed by heart failure (risk increased by 3.27) and osteoporosis (without current pathological fracture, risk increased by 2.75 and for osteoporosis with current pathological fracture, risk increased by 2.69) (Table 2).

**Table 2:** Percentage of patients with comorbidities in the case-control population (2 years before and after index date)

Comorbidities	COPD patients (*n* = 17,479)	Reference population (*n* = 84,514)	Relative risk ratio and *p*-value
*Heart failure*			
Heart failure-I50	5350 (30.61)	7899 (9.35)	3.27 (95% CI 3.17–3.37), *p* < 0.0001
Hypertensive heart disease-I11	456 (2.61)	903 (1.07)	2.44 (95% CI 2.18–2.73), *p* < 0.0001
*Ischemic heart disease*			
Angina pectoris-I20	3308 (18.93)	7949 (9.41)	2.01 (95% CI 1.94–2.09), *p* < 0.0001
ST elevation (STEMI) and non-ST elevation (NSTEMI) myocardial infarction-I21	2338 (13.38)	5026 (5.95)	2.25 (95% CI 2.15–2.35), *p* < 0.0001
Chronic ischemic heart disease-I25	3448 (19.73)	7048 (8.35)	2.36 (95% CI 2.28–2.45), *p* < 0.0001
*Hypertension*			
Essential (primary) hypertension-I10	9838 (56.28)	31,675 (37.51)	1.50 (95% CI 1.48–1.52), *p* < 0.0001
*Depression/anxiety*			
Major depressive disorder, single episode-F32	3368 (19.27)	8137 (9.63)	2.00 (95% CI 1.93–2.07), *p* < 0.0001
Major depressive disorder, recurrent-F33	761 (4.35)	1676 (1.98)	2.19 (95% CI 2.02–2.39), *p* < 0.0001
Other anxiety disorders-F41	2500 (14.30)	5523 (6.54)	2.19 (95% CI 2.09–2.29), *p* < 0.0001
*Sleep disorders*			
Sleep disorders-G47	1439 (8.23)	4039 (4.78)	1.72 (95% CI 1.62–1.82), *p* < 0.0001
*Osteoporosis*			
Osteoporosis with current pathological fracture-M80	809 (4.63)	1452 (1.72)	2.69 (95% CI 2.47–2.93), *p* < 0.0001
Osteoporosis without current pathological fracture-M81	1450 (8.30)	2552 (3.02)	2.75 (95% CI 2.58–2.92), *p* < 0.0001
*Lung cancer*			
Malignant neoplasm of bronchus and lung-C34	1091 (6.24)	883 (1.05)	5.97 (95% CI 5.47–6.51), *p* < 0.0001
*Stroke*			
Cerebral infarction-I63	1782 (10.20)	5047 (5.98)	1.71 (95% CI 1.62–1.80), *p* < 0.0001
Cerebral infarction-I64	524 (3.00)	1496 (1.77)	1.69 (95% CI 1.53–1.87), *p* < 0.0001
*Osteoarthritis*			
Osteoarthritis of hip-M16	1400 (8.01)	4710 (5.58)	1.44 (95% CI 1.36–1.52), *p* < 0.0001
Osteoarthritis of knee-M17	1803 (10.32)	8573 (10.15)	1.02 (95% CI 0.97–1.07), *p* = 0.5037
Osteoarthritis of first carpometacarpal joint-M18	443 (2.53)	1607 (1.90)	1.33 (95% CI 1.20–1.48), *p* < 0.0001
Other and unspecified osteoarthritis-M19	1730 (9.90)	6331 (7.50)	1.32 (95% CI 1.26–1.39), *p* < 0.0001
*Others*			
Pulmonary embolism-I26	729 (4.17)	1471 (1.74)	2.39 (95% CI 2.19–2.61), *p* < 0.0001
Phlebitis and thrombophlebitis-I80	1140 (6.52)	3450 (4.09)	1.60 (95% CI 1.50–1.70), *p* < 0.0001

ICD-10 codes mentioned alongside comorbidities

*CI* confidence interval, *COPD* chronic obstructive pulmonary disease, *ICD-10* International Classification of Diseases, tenth revision, *NSTEMI* non-ST-elevation myocardial infarction, *STEMI* ST-elevation myocardial infarction

### Mortality

Comorbidities that led to significantly increased mortality in the COPD population than the reference population included ischemic heart disease, hypertension, depression, anxiety, sleep disorders, osteoporosis, osteoarthritis, and asthma. The highest risk of mortality was observed with sleep disorders (hazard ratio [HR]: 1.85, 95% confidence interval [CI]: 1.34–2.56, *p* = 0.0002), followed by osteoarthritis (HR: 1.67, 95% CI: 1.44–1.93, *p* < 0.0001) (Table 3).

**Table 3:** Risk of mortality in COPD vs. reference populations with same comorbidities

Comorbidities	Hazard ratio (95% CI)	*p*-value
Heart failure	1.04 (95% CI 0.97–1.13)	0.2503
Ischemic heart disease	1.25 (95% CI 1.16–1.35)	<0.0001
Hypertension	1.54 (95% CI 1.45–1.63)	<0.0001
Depression	1.22 (95% CI 1.04–1.43)	0.0118
Anxiety	1.52 (95% CI 1.23–1.87)	<0.0001
Sleep disorders	1.85 (95% CI 1.34–2.56)	0.0002
Osteoporosis	1.53 (95% CI 1.27–1.83)	<0.0001
Lung cancer	0.69 (95% CI 0.56–0.83)	0.0001
Stroke	1.02 (95% CI 0.89–1.18)	0.7444
Osteoarthritis	1.67 (95% CI 1.44–1.93)	<0.0001
Asthma	1.63 (95% CI 1.47–1.81)	<0.0001

Hazard ratios reported for time to death between the two groups i.e., COPD with comorbidity vs. reference population with comorbidity

*CI* confidence interval, *COPD* chronic obstructive pulmonary disease

### Exacerbations

Further, time to first exacerbation (any) in COPD patients was significantly decreased with the presence of the following comorbidities: heart failure, ischemic heart disease, hypertension, anxiety, osteoporosis, lung cancer, stroke, and asthma compared with COPD patients without these comorbidities (Table S4 in the additional file). Presence of lung cancer significantly decreased the time to first exacerbation, with a much larger magnitude compared with other comorbidities (HR: 1.56, 95% CI: 1.40–1.73, *p* < 0.0001), followed by heart failure (HR: 1.40, 95% CI: 1.33–1.47, *p* < 0.0001) (Table S4 in the additional file).

Comorbidities that were observed to be significantly associated with increased number of severe exacerbations in COPD patients included heart failure (rate ratio: 2.69, 95% CI, 2.61–2.77), ischemic heart disease (1.88, 95% CI: 1.83–1.93), depression/anxiety (1.44, 95% CI: 1.40–1.48), sleep disorders (1.26, 95% CI: 1.23–1.29), osteoporosis (1.53, 95% CI: 1.48–1.58), lung cancer (1.32, 95% CI: 1.17–1.47), and stroke (1.47, 95% CI: 1.42–1.52) (Table 4). Osteoarthritis was associated with lower number of severe exacerbations and hypertension had no effect on the number of severe exacerbations.

**Table 4:** Concurrent comorbidities and their association with the number of severe exacerbations

Comorbidities	Mean number of severe exacerbations/year	Rate ratio, *p*-values
*Heart failure*		
With disease, *n* = 5158	0.35 (95% CI 0.34–0.36)	2.69 (95% CI 2.61–2.77), *p* < 0.0001
Without disease, *n* = 12,321	0.13 (95% CI 0.12–0.14)	
*Ischemic heart disease*		
With disease, *n* = 4690	0.30 (95% CI 0.29–0.31)	1.88 (95% CI 1.83–1.93), *p* < 0.0001
Without disease, *n* = 12,789	0.16 (95% CI 0.15–0.16)	
*Hypertension*		
With disease, *n* = 9291	0.20 (95% CI 0.19–0.21)	1.05 (95% CI 1.03–1.07), *p* = 0.1288
Without disease, *n* = 8188	0.19 (95% CI 0.18–0.20)	
*Depression/anxiety*		
With disease, *n* = 3699	0.26 (95% CI 0.25–0.27)	1.44 (95% CI 1.40–1.48), *p* < 0.0001
Without disease, *n* = 13,780	0.18 (95% CI 0.17–0.19)	
*Sleep disorders*		
With disease, *n* = 1174	0.24 (95% CI 0.22–0.26)	1.26 (95% CI 1.23–1.29), *p* = 0.0002
Without disease, *n* = 16,305	0.19 (95% CI 0.19–0.20)	
Osteoporosis		
With disease, *N* = 1553	0.29 (95% CI 0.27–0.31)	1.53 (95% CI 1.48–1.58), *p* < 0.0001
Without disease, *N* = 15,926	0.19 (95% CI 0.18–0.19)	
*Lung cancer*		
With disease, *n* = 1052	0.25 (95% CI 0.22–0.27)	1.32 (95% CI 1.17–1.47), *p* < 0.0001
Without disease, *n* = 16,427	0.19 (95% CI 0.19–0.20)	
*Stroke*		
With disease, *n* = 1687	0.28 (95% CI 0.26–0.30)	1.47 (95% CI 1.42–1.52), *p* < 0.0001
Without disease, *n* = 15,792	0.19 (95% CI 0.18–0.19)	
*Osteoarthritis*		
With disease, *n* = 2929	0.17 (95% CI 0.16–0.19)	0.85 (95% CI 0.83–0.87), *p* = 0.0015
Without disease, *n* = 14,550	0.20 (95% CI 0.19–0.21)	
*Asthma*		
With disease, *n* = 4924	0.22 (95% CI 0.20–0.23)	1.16 (95% CI 1.12–1.20), *p* = < 0.0001
Without disease, *n* = 12.555	0.19 (95% CI 0.18–0.20)	

*CI* confidence interval

### Costs

**Fig. 2:**
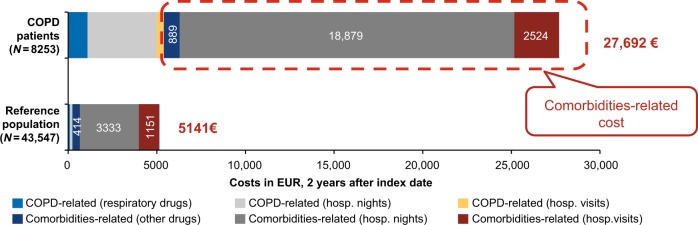
HCRU in COPD patients vs. reference population-cumulative costs of 2 years after the index date, 2013, For the reference population, COPD-related costs include respiratory drugs mainly for asthma, COPD chronic obstructive pulmonary disease; EUR Euro; HCRU healthcare resource utilization

The cumulative HCRU costs over 2 years after the index date associated with comorbidities were significantly higher in COPD patients (€27,692) compared with the reference group (€5141) (*p* < 0.0001) (Fig. 2). Comorbidities accounted for >80% of costs in COPD patients (€22,292), with costs associated with hospital nights as the major contributor (€18,879) when calculated 2 years after the index date (Fig. 2).

**Fig. 3:**
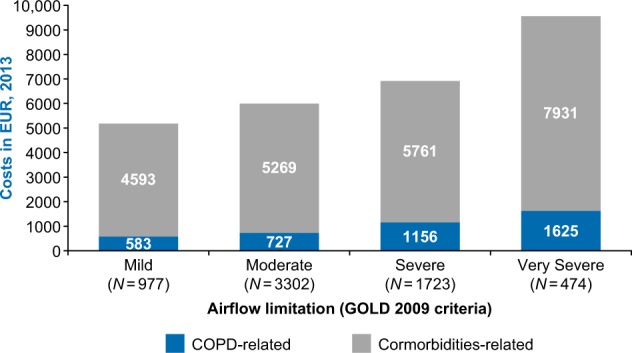
HCRU in COPD patients based on severity strata cohort COPD chronic obstructive pulmonary disease; GOLD Global Initiative for Chronic Obstructive Lung Disease; HCRU healthcare resource utilization

Comorbidity-related costs were positively correlated with disease severity (mild vs. very severe accounted for €4593 vs. €7931, respectively) when analyzed in the COPD severity strata cohort (Fig. 3).

COPD patients aged between 60 and 65 years had the highest total costs associated with comorbidities, including sleep disorders (€41,751/year; 43 times), osteoporosis (€50,234/year; eight times), and heart failure (€36,465/year; three times) compared with the reference population with these comorbidities (Table S5 in the additional file).

### Presence of more than one comorbidity

COPD patients with more than one comorbidity showed worse outcomes than those with only one comorbidity. COPD patients with both heart failure and diabetes (*n* = 3812), and both heart failure and angina (*n* = 3264) showed a considerably increased risk of mortality than the reference population with these comorbidities (*N* = 5661 and *N* = 3035, respectively) (HR: 1.281, 95% CI: 1.207–1.359, *p* < 0.0001; HR: 1.167, 95% CI: 1.094–1.246, *p* < 0.0001, respectively). Further, the time to first exacerbation was significantly decreased in COPD patients with both heart failure and diabetes, and both heart failure and angina compared to the COPD population without these comorbidities (HR: 1.306, 95% CI: 1.255–1.360, *p* < 0.0001; HR: 1.354, 95% CI: 1.297–1.414, *p* < 0.0001, respectively). However, it was observed that having three comorbidities did not further increase the risk of mortality and exacerbations in COPD patients.

## Discussion

Main findings from this large, real-world retrospective study were that Swedish primary care patients with COPD had significantly higher prevalence of comorbidities compared with a non-COPD population. Prevalence rates of lung cancer, heart failure, ischemic heart disease, hypertension, depression/anxiety, sleep disorders, osteoporosis, stroke, osteoarthritis, and pulmonary embolism, were significantly higher in the COPD population compared with the non-COPD population. After 2 years post-diagnosis, the highest percentage increase was observed in the prevalence of cardiovascular diseases, which was 81.8% in COPD patients compared to 30.7% in the non-COPD population; this observation was in alignment with findings from other studies.^12,14,15,20^ Although aging increases the risk of developing several comorbidities,^21^ results from this study suggest that the presence of COPD significantly exaggerates the risk of such comorbidities in elderly patients.

Our results also indicated that these comorbidities have a high impact on the occurrence of severe exacerbations and mortality in COPD patients. It was found that COPD patients with heart failure, ischemic heart disease, depression/anxiety, sleep disorders, osteoporosis, lung cancer, and stroke were significantly more prone to have severe exacerbations than patients without these comorbidities. The reason why patients with osteoarthritis had fewer severe exacerbations remains unclear. Further, COPD patients with ischemic heart disease, hypertension, depression, anxiety, sleep disorders, osteoporosis, osteoarthritis, and asthma had a significantly higher risk of death than the non-COPD population. Research is needed though to establish cause of association of these comorbidities with the increased mortality rates in COPD patients. Surprisingly, a significantly lower risk of mortality was observed in COPD patients with lung cancer vs. the non-COPD population with lung cancer (HR: 0.69, 95% CI: 0.56–0.83, *p* = 0.0001). This might be due to earlier detection of lung cancer in COPD patients than in the non-COPD population as physicians expect lung cancer in COPD patients and often recommend them for X-ray imaging. The finding that relatively more frequent exacerbations and increased mortality were observed in COPD patients with more than one comorbidity indicates that exacerbations can also be prevented through proper management of comorbidities.

The cumulative healthcare costs associated with comorbidities were observed to be more than five times higher in the COPD population compared with the non-COPD population. Healthcare costs associated with these comorbidities were further found to increase with COPD severity.

The strength of this study is based on its large sample size, which comprised patients across the whole-COPD classification spectrum. Further, primary care setting and real-world study design represent the general population and clinical practice in Sweden. The study carried out a comprehensive assessment of comorbidities present in COPD patients and their association with exacerbations, mortality, and costs. Data quantification in this study allows decision-makers to identify-specific comorbidities that markedly increase burden among COPD patients and analyze how integrated screening and treatment strategies can be devised in such patients, thereby aiming to reduce the associated burden.

Results from this study are in agreement with other published studies;^4–10,12–18,22–25^ however, they provide more clear differentiation in terms of the risk association with exacerbations, mortality, and healthcare costs.

The study also has certain limitations. Because of the retrospective design of the study, the analysis could be affected by unobserved differences among patients. The reference population was identified from the study primary care centers rather than from the general population. Underestimation of comorbidities might have occurred, which is common in the elderly population. Further, the findings from this study are only limited to Sweden and cannot be generalized to populations applicable to other healthcare systems.

Nevertheless, the findings suggest that proper characterization of COPD patients needs to be carried out in terms of comorbidities as these are important prognostic variables that can significantly contribute to the increased morbidity and mortality in COPD patients. In addition, the results suggest the importance of patient-centered treatment strategies and the targeted allocation of healthcare resources in order to manage the associated humanistic and economic burden.^26,27^

In conclusion, the present study demonstrated a significantly increased prevalence of comorbidities in the COPD population than the non-COPD population. These comorbidities contribute to substantial morbidity, mortality, and economic burden in COPD patients. Therefore, these comorbidities should be comprehensively evaluated and considered while making clinical decisions in COPD patients.

## Methods

### Study design

ARCTIC was a large, real-world, retrospective Swedish cohort study conducted in 18,586 eligible primary care COPD patients.

Electronic medical record (EMR) data collected between 2000 and 2014 for COPD patients from 52 primary care centers across Sweden using an established software system (Pygargus Customized eXtraction Program [CXP]) included age and gender, prescriptions (according to the World Health Organization Anatomic Therapeutic Chemical [ATC] codes), diagnoses (according to the International Classification of Diseases, tenth revision [ICD-10] codes), spirometry measurements, laboratory tests, healthcare professional (HCP) visits, and referrals. The centers covered urban and rural sites of varying sizes across Sweden. EMR data were linked using unique individual pseudonymized identification numbers to National Registry data sources. These included (i) the Longitudinal Integration Database for Health Insurance and Labor Market Studies, which contains socio-demographic data, including educational level, marital status and family situation, occupational status, retirement, and economic compensation and social benefits; (ii) the National Patient Register, which contains data relating to diagnosis from secondary care (ICD-10 code and associated position), including surgery, gender, age, region, hospital visits, specialty visits, hospital admissions, discharges, medical procedures, and surgeries performed in inpatient and outpatient specialist settings; (iii) the National Prescription Register, which contains the full details of all dispensed medications (ATC codes) from both primary and secondary care, including brand name, prescription date, dose, strength, pack size, and specialty of the prescriber and costs associated with the drug prescription; and (iv) the Cause of Death Register, which holds information relating to sex, date of death, and the underlying cause of death.

### Study patients

The study included patients aged ≥40 years who had received either a physician’s diagnosis of COPD (ICD-10 code: J44) in primary care (EMR database) or a physician’s diagnosis of asthma (ICD-10 code: J45/J46) in primary care that was later verified as COPD, or COPD was added to the asthma diagnosis in the hospital setting according to the National Patient Register. The index date was the time of the first recorded physician’s diagnosis of COPD during the enrollment timeframe. A non-COPD reference population was identified at each center for age, sex, and matching comorbidities.

### Outcomes

Various comorbidities assessed in the study are mentioned in the additional file. Chronic bronchitis was also analyzed; however, because of uncertainties in the usage of its diagnosis code, it was excluded. The comorbidity groups were created using comorbidity information at the COPD index date plus 6 months, since several comorbidities are diagnosed around the COPD index date.

Comorbidity prevalence (%) of each disease at 2 years pre- and post-first COPD diagnosis and the associated impact on HCRU costs, including costs of hospital visits, hospital nights, and drugs were evaluated between COPD patients and non-COPD patients (reference population). Hospital nights were the sum of overnight stays at the hospital per patient. Hospitalization events were the number of times that a patient had been in a ward at a hospital. A visit to a hospital where the patients had not stayed overnight was considered to be a hospital visit. Costs were converted to the year 2013, and a conversion factor of 8/1 was used from Swedish Krona (SEK) to Euro (€). Further, the association of these comorbidities with mortality and exacerbations was assessed. Exacerbations were defined as COPD-related hospitalizations (J44 in primary position or J44.0/J44.1 in secondary position), emergency visits (J44.0/J44.1 in outpatient hospital care), or collection of oral steroids (ATC H02AB) or antibiotics targeted at respiratory diseases (ATC J01AA/J01CA). Exacerbations occurring within 14 days were considered as one single event. Severe exacerbations were defined as hospitalizations due to COPD exacerbation in secondary care (ICD-10: J44) and/or emergency visits to a hospital for COPD (J44.1). Recurrent exacerbations occurring within 14 days were considered as one unique event. Within the COPD group, comorbidity-related costs were also analyzed based on the severity strata cohort (airflow limitation classified per the Global Initiative for Chronic Obstructive Lung Disease [GOLD] guidelines 2009: mild: FEV_1_ ≥80% predicted, moderate: 50% ≤FEV_1_ <80% predicted, severe: 30% ≤FEV_1_ <50% predicted, and very severe: FEV_1_ <30% predicted or FEV_1_ <50% predicted plus chronic respiratory failure) and various age groups.

### Statistical analysis

The time from the index date to the first exacerbation and time to death were compared between the comorbidity groups using Cox regression with time to event as the dependent variable, the comorbidity group as the independent variable, and age and gender as covariates. Hazard ratios between the groups were calculated along with *p*-values, comparing the differences between the groups. The effect of COPD on mortality was quantified by comparing the COPD population and the reference population for patients with the same comorbidity. Case-control cohorts were created for each comorbidity group between the COPD population and the reference population by matching age, sex, index year, and length of comorbidity duration. The index year was included to compensate for improved survival during the time period covered. An analysis of covariance was used to assess the association of comorbidities with the increased number of severe exacerbations. The number of severe exacerbations per year was accounted as the dependent variable, the comorbidity group as the independent variable, and age and gender as covariates. Indirect costs (defined as loss of income due to early retirement and sick leaves) were calculated for the COPD patients and compared across comorbidities. Differences between groups with and without the comorbidity were calculated and compared using an analysis of covariance with indirect cost as the dependent variable, groups with and without the comorbidity as independent variables, and age and gender as covariates.

Ethical approval for the original study was obtained from the local Ethical Regional Board in Uppsala, Sweden, on December 11, 2014 (number: 2014–397) for accessing the national health register and for recruiting primary care centers to the study. An amendment specifying additional analysis was approved by the Ethical Regional Board in Uppsala on October 6, 2017. All data were de-identified, and therefore patient consent was not required by the ethics committee.

## Electronic supplementary material

Supplementary material

## Data Availability

The authors declare that data supporting the findings of this study are available within the paper and the additional file.

## Electronic supplementary material

**Supplementary information** accompanies the paper on the *npj Primary Care Respiratory Medicine* website (10.1038/s41533-018-0101-y).
