# Supplementary material for: Real-world retrospective cohort study ARCTIC shows burden of comorbidities in Swedish COPD versus non-COPD patients
Source: NPJ Prim Care Respir Med. 2018 Sep 10;28:33. doi: 10.1038/s41533-018-0101-y (PMC6131165; doi:10.1038/s41533-018-0101-y)
Supplement: Supplementary file 1 — Supplementary material [file 41533_2018_101_MOESM1_ESM.pdf]

Methods

Outcomes: Following comorbidities defined through diagnosis codes in the primary or secondary care setting were assessed in the study:

Asthma
Cardiovascular disease (including ischemic heart disease, arrhythmias, heart failure, stroke, and peripheral vascular disease)
Diabetes (both type I and type II)
Hyperlipidemia
Depression, Anxiety
Gastro-esophageal reflux disease
Chronic pain
Cognitive impairment/dementia
Osteoporosis/Fractures
Lung cancer
Any other cancer
Rhinitis
Nasal polyps
Polymyalgia rheumatic
Kidney disease
Rheumatoid arthritis

It was hypothesized that some comorbidities were underreported when evaluated through registered diagnosis codes. Accordingly, following comorbidities were also identified through the use of medication according to the National Prescription Register:

Cardiovascular medication (for conditions mentioned above)
Antihypertensive medication
Medication against cognitive impairment (Alzheimer's)
Proton-pump inhibitors
Antidepressants
Statins
Central pain killers
Sleep medication
Bisphosphonates
Beta-blockers
Nasal corticosteroids
Diabetes medication

In addition, the following comorbidities (events related to inhaled corticosteroid use) were defined through diagnosis codes/injury codes in primary or secondary care:

Pneumonia
Infection (Tuberculosis, Lower respiratory tract infections (including Acute bronchitis) and Urinary tract infection)
Cataracts
Oral candida infection
Osteoporosis/Fractures
Gastric and Duodenal ulcers

Results

Table S1 Percent of patients with comorbidities in case-control population (>5 fold increase in COPD; 2 years before and after index date)

Disease	COPD population	Reference population	Factor increased
Emphysema - J43	1400 (8.01)	168 (0.20)	40.05
Respiratory failure, not elsewhere classified - J96	1944 (11.12)	559 (0.66)	16.85
Unspecified chronic bronchitis - J42	1027 (5.88)	404 (0.48)	12.25
Surgical operation and other surgical procedures as the cause of abnormal reaction of the patient, or of later complication, without mention of misadventure at the time of the procedure - Y83	638 (3.65)	267 (0.32)	11.41
Unspecified fall - W19	564 (3.23)	267 (0.32)	10.09
Problems related to lifestyle - Z72	1332 (7.62)	693 (0.82)	9.29
Other pulmonary heart diseases - I27	423 (2.42)	231 (0.27)	8.96
Fall on same level from slipping, tripping and stumbling - W01	1175 (6.72)	694 (0.82)	8.2
Nicotine dependence - F17	2214 (12.67)	1310 (1.55)	8.17
Abnormal findings on diagnostic imaging of lung - R91	1276 (7.30)	994 (1.18)	6.19
Malignant neoplasm of bronchus and lung - C34	1091 (6.24)	883 (1.05)	5.94
Other respiratory disorders - J98	538 (3.08)	458 (0.54)	5.7
Dependence on enabling machines and devices, not elsewhere classified - Z99	494 (2.83)	473 (0.56)	5.05

COPD, chronic obstructive pulmonary disease

Table S2 Percent of patients with comorbidities in case-control population (>3 fold increase in COPD; 2 years before and after index date)

Disease	COPD population	Reference population	Factor increased
Neoplasm of uncertain behavior of middle ear and respiratory and intrathoracic organs - D38	854 (4.89)	902 (1.07)	4.57
Bacterial pneumonia, not elsewhere classified - J15	1904 (10.89)	2047 (2.42)	4.5
Aortic aneurysm and dissection - I71	825 (4.72)	937 (1.11)	4.25
Streptococcus, Staphylococcus, and Enterococcus as the cause of diseases classified elsewhere - B95	594 (3.40)	708 (0.84)	4.05
Other bacterial agents as the cause of diseases classified elsewhere - B96	1024 (5.86)	1261 (1.49)	3.93
Occupant of heavy transport vehicle injured in non-collision transport accident - V68	533 (3.05)	668 (0.79)	3.86
Poisoning by, adverse effect of and under dosing of diuretics and other and unspecified drugs, medicaments and biological substances - T50	501 (2.87)	662 (0.78)	3.68
Personal history of certain other diseases - Z86	1425 (8.15)	1881 (2.23)	3.65

Disease	COPD population	Reference population	Factor increased
Acute bronchitis - J20	1856 (10.62)	2464 (2.92)	3.64
Symptoms and signs concerning food and fluid intake - R63	553 (3.16)	736 (0.87)	3.63
Other peripheral vascular diseases - I73	1159 (6.63)	1582 (1.87)	3.55
Heart failure - I50	5350 (30.61)	7899 (9.35)	3.27
Pneumonia, unspecified organism - J18	5462 (31.25)	8304 (9.83)	3.18
Abnormalities of breathing - R06	4767 (27.27)	7410 (8.77)	3.11
Chronic kidney disease - N18	1031 (5.90)	1647 (1.95)	3.03
Volume depletion - E86	787 (4.50)	1261 (1.49)	3.02
Encounter for fitting and adjustment of other devices - Z46	470 (2.69)	752 (0.89)	3.02
Other disorders of fluid, electrolyte and acid-base balance - E87	1310 (7.49)	2101 (2.49)	3.01

COPD, chronic obstructive pulmonary disease

Table S3 Percent of patients with comorbidities in case-control population (>2.5 fold increase in COPD; 2 years before and after index date)

Disease	COPD population	Reference population	Factor increased
Acute kidney failure - N17	689 (3.94)	1115 (1.32)	2.98
Atherosclerosis - I70	1301 (7.44)	2147 (2.54)	2.93
Non-pressure chronic ulcer of lower limb, not elsewhere classified - L97	795 (4.55)	1368 (1.62)	2.81
Pleural effusion, not elsewhere classified - J90	621 (3.55)	1078 (1.28)	2.77
Osteoporosis without current pathological fracture - M81	1450 (8.30)	2552 (3.02)	2.75
Unspecified kidney failure - N19	599 (3.43)	1055 (1.25)	2.74
Viral and other specified intestinal infections - A08	431 (2.47)	759 (0.90)	2.74
Edema, not elsewhere classified - R60	1587 (9.08)	2805 (3.32)	2.73
Alcohol related disorders - F10	1741 (9.96)	3098 (3.67)	2.71
Osteoporosis with current pathological fracture - M80	809 (4.63)	1452 (1.72)	2.69
Occlusion and stenosis of pre-cerebral arteries, not resulting in cerebral infarction - I65	504 (2.88)	922 (1.09)	2.64
Other complications of surgical and medical care, not elsewhere classified - T88	582 (3.33)	1107 (1.31)	2.54
Esophagitis - K20	444 (2.54)	846 (1.00)	2.54
Unspecified acute lower respiratory infection - J22	3634 (20.79)	7016 (8.31)	2.5

COPD, chronic obstructive pulmonary disease

Table S4 Time to first exacerbation (any) with the presence of comorbidities vs. no comorbidities in COPD patients

Comorbidity in COPD patients	Hazard ratio (95% CI)	p-value
Heart failure	1.40 (95% CI 1.33 :1.47)	<.0001
Ischemic heart disease	1.26 (95% CI 1.20 :1.32)	<.0001
Hypertension	1.10 (95% CI 1.07 :1.14)	<.0001
Depression	1.06 (95% CI 0.99 :1.14)	0.0913
Anxiety	1.10 (95% CI 1.01 :1.19)	0.0342
Sleep disorders	1.04 (95% CI 0.92 :1.16)	0.5557
Osteoporosis	1.12 (95% CI 1.02 :1.24)	0.0151
Lung cancer	1.56 (95% CI 1.40 :1.73)	<.0001
Stroke	1.33 (95% CI 1.21 :1.47)	<.0001
Osteoarthritis	1.00 (95% CI 0.93 :1.07)	0.9360
Asthma	1.18 (95% CI 1.13 :1.23)	<.0001

CI, confidence interval; COPD, chronic obstructive pulmonary disease

Table S5 Direct and indirect costs in relation to comorbidities in patients aged 60-65 years (€/year)

Comorbidity	COPD with comorbidity	Reference with comorbidity	Difference in costs (fold increase)
**Total costs**			
Osteoporosis	57,853 (n=151)	7,619 (n=293)	50,234 (8×)
Heart failure	56,804 (n=259)	20,339 (n=394)	36,465 (3×)
Sleep disorders	42,737 (n=207)	986 (n=780)	41,751 (43×)
**Direct costs**			
Osteoporosis	44,617 (n=198)	7,295 (n=319)	37,322 (6×)
Heart failure	39,620 (n=432)	12,864 (n=532)	26,756 (3×)
Sleep disorders	31,734 (n=248)	986 (n=818)	30,748 (32×)
**Indirect costs**			
Osteoporosis	13,236 (n=151)	NA	NA
Heart failure	17,184 (n=259)	NA	NA
Sleep disorders	11,003 (n=207)	NA	NA

COPD, chronic obstructive pulmonary disease; NA, not available
